# LICSS - a chemical spreadsheet in microsoft excel

**DOI:** 10.1186/1758-2946-4-3

**Published:** 2012-02-02

**Authors:** Kevin R Lawson, Jonty Lawson

**Affiliations:** 1 Syngenta Ltd. Jealotts Hill Research Centre, Bracknell, Berkshire, RG42 6EY, UK; 2 DataLook Ltd. Highfield, Preston, PR25 5SD, UK

## Abstract

**Background:**

Representations of chemical datasets in spreadsheet format are important for ready data assimilation and manipulation. In addition to the normal spreadsheet facilities, chemical spreadsheets need to have visualisable chemical structures and data searchable by chemical as well as textual queries. Many such chemical spreadsheet tools are available, some operating in the familiar Microsoft Excel environment. However, within this group, the performance of Excel is often compromised, particularly in terms of the number of compounds which can usefully be stored on a sheet.

**Summary:**

LICSS is a lightweight chemical spreadsheet within Microsoft Excel for Windows. LICSS stores structures solely as Smiles strings. Chemical operations are carried out by calling Java code modules which use the CDK, JChemPaint and OPSIN libraries to provide cheminformatics functionality. Compounds in sheets or charts may be visualised (individually or *en masse*), and sheets may be searched by substructure or similarity. All the molecular descriptors available in CDK may be calculated for compounds (in batch or on-the-fly), and various cheminformatic operations such as fingerprint calculation, Sammon mapping, clustering and R group table creation may be carried out.

We detail here the features of LICSS and how they are implemented. We also explain the design criteria, particularly in terms of potential corporate use, which led to this particular implementation.

**Conclusions:**

LICSS is an Excel-based chemical spreadsheet with a difference:

• It can usefully be used on sheets containing hundreds of thousands of compounds; it doesn't compromise the normal performance of Microsoft Excel

• It is designed to be installed and run in environments in which users do not have admin privileges; installation involves merely file copying, and sharing of LICSS sheets invokes automatic installation

• It is free and extensible

LICSS is open source software and we hope sufficient detail is provided here to enable developers to add their own features and share with the community.

## Introduction

The familiar Chemical Spreadsheet paradigm is an extremely useful way of presenting structural information together with calculated or measured structural properties. Indeed, most software which handles or stores chemical data will make available a tabular view implementing at least some of the more common spreadsheet functionality such as sorting by columns. Many excellent chemical spreadsheet tools are commercially available and there are also notable freeware/open source examples [1]. Most such software is self-contained which, of course, gives the developers maximum freedom of implementation. This approach has certain potential disadvantages however, particularly considered in the context of a corporate environment:

• An interested user needs to buy/download and install the software. This of course is trivial in the case of a 'home' or independent user but may pose almost insurmountable challenges in a 'locked-down' corporate environment

• The user must get to grips with an entirely new piece of software overcoming a potentially steep learning curve

• It is extremely difficult to provide spreadsheet features (powerful calculated columns, visualisation, macro language, etc) which begin to rival those of the industry standard, Microsoft Excel - a program already very familiar to target users.

The last point suggests a different approach in which the chemistry engine is build on top of Excel. This tactic appears extremely attractive partly because the potential developer can concentrate on implementing chemical functionality but also because of the ubiquity and power of Excel. Two well-known realisations of this approach are Isis for Excel [2] and Accord for Excel [3].

Solutions of this type are typically implemented as Excel AddIns, using Visual Basic for Applications (VBA) to interface with chemistry engines. Structures are usually stored on the spreadsheets as some kind of object (including structure-layout or image data) which may be interpreted by the chemistry engine for visualisation and calculation purposes. To ensure that structure objects display and sort properly, it is usually necessary to intercept several of Excel's fundamental calls (such as the main calculation routine). This necessity, together with the size of the stored objects, can lead to rapid degradation of performance for spreadsheets containing large numbers of structures.

Bearing the foregoing in mind, LICSS was designed to appeal particularly to corporate users of Excel for Windows. Because of one of the authors' experience of corporate locked-down environments and because LICSS was to be a 'hobby' project, initially with just one spare-time developer, some rather specific design criteria were developed:

• LICSS should require no installation beyond file copying. Users should be able to share spreadsheets with fully automatic installation (if necessary)

• LICSS would implement chemistry functionality by interfacing with the excellent CDK Java library [4,5] (and the corresponding rendering package, JChemPaint [6])

• Structures should be stored purely as Smiles strings in cells; structure rendering would be on-the-fly

• LICSS spreadsheets would not intercept Excel's calculation calls

• An Excel add-in would not be used (they normally need user installation and can require admin privileges). Any necessary VBA would exist on each chemically-enabled spreadsheet.

### User Implementation and Features

**Figure 1:**
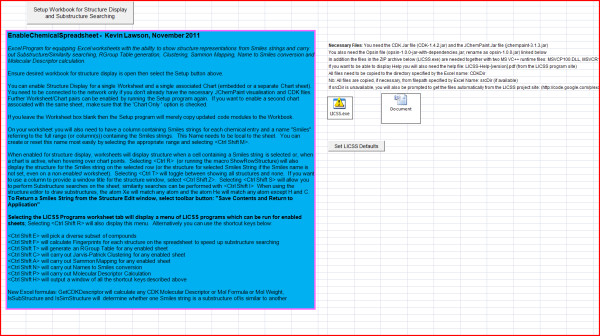
**EnableChemicalSpreadsheetV2.1.xls**. Choosing the "Select Workbook for Structure Display and Substructure Searching" button will present user with a dropdown listing currently open workbooks together with their associated worksheets and charts which may be chemically-enabled.

From a user's point of view, LICSS is implemented as a single Excel for Windows workbook with just one routine which allows chemical enabling of any suitable spreadsheets (containing Smiles strings) and associated charts (Figure 1). Once enabled, the spreadsheets are entirely standalone, requiring no add-ins or any customisation of Excel [7]. If shared with other users, or moved to a workstation without LICSS installation, the enabled sheets install LICSS seamlessly (if available in some shared area) or, if necessary, prompt the user to allow automatic file install from the LICSS project site on Google projects [8].

**Figure 2:**
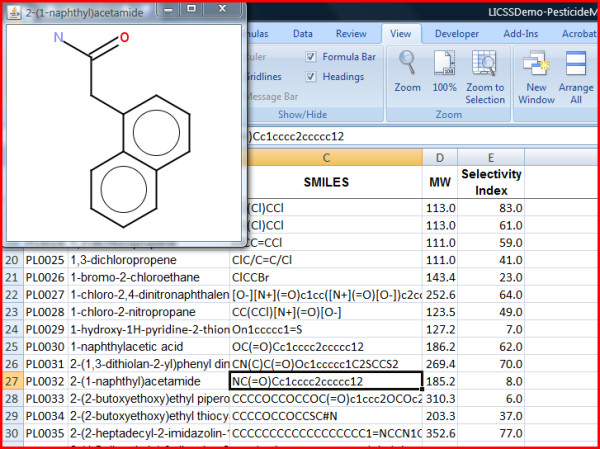
**LICSS display of single compounds upon selecting cells from the Smiles column**.

**Figure 3:**
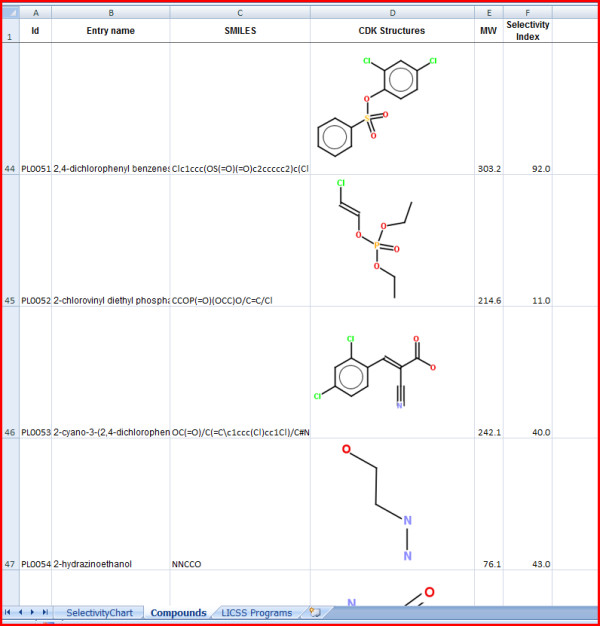
**Display of multiple compounds in LICSS sheets**.

LICSS-enabled sheets use JChemPaint to render Smiles strings in a pop-up window (Figure 2). This is activated by clicking directly on the Smiles string, choosing a shortcut key to show the first structure on a row, or by mouse hover over scatter chart data points. If desired, users can also choose to display structures for all visible cells (Figure 3). The routine which achieves this calculates only which cells are currently visible to the user and renders the structures for them on-the-fly. This method ensures that even very large sheets (> 100,000 compounds) may be visualised without running out of memory.

**Figure 4:**
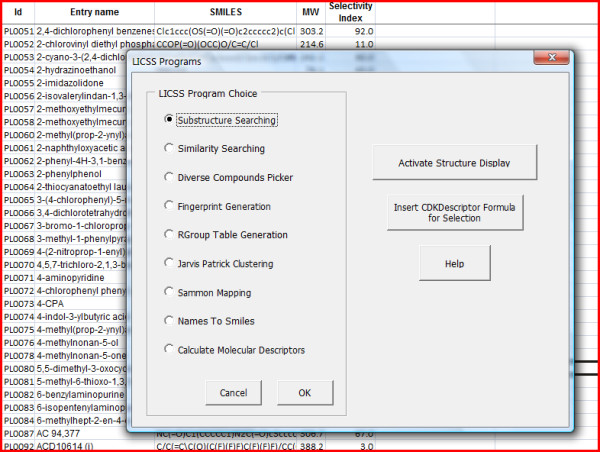
**LICSS main programs menu**.

Clicking on the 'LICSS Programs' worksheet tab gives access to a single menu making all other LICSS functionality available (Figure 4).

**Figure 5:**
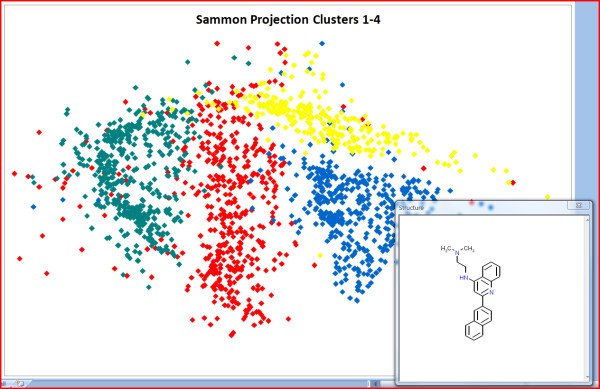
**Sammon Projection of part of the Welcome Anti-Malarials data set calculated by LICSS**. Single compound display on hovering over chart data points is also shown.

Routines are currently available for substructure and similarity searching, fingerprint generation (for faster substructure searching), R Group table generation, Jarvis-Patrick clustering, Sammon map coordinate generation (see Figure 5 for a scatter plot created from LICSS-generated Sammon map coordinates), diverse compound picking, molecular descriptor calculation and conversion of IUPAC names to Smiles (using the OPSIN Java library [9]). New Excel formulas are also available - for calculating molecular descriptors, molecular weight or molecular formula and for determining whether one Smiles string is a substructure of, or is similar to, another Smiles string (within a defined threshold). Table 1 gives some indicative data for the performance a user can expect from LICSS functionality.

**Table 1:** Timings for common cheminformatics tasks using LICSS.

Dataset	Operation	Timing (m:s)	Hits
[1]	SSS (Sub Structure Search) with n1cnccc1 (Smarts matching)	0:13	76

[1]	SSS with pyrimidine (sketcher)	0:05	76

[1]	SSS with n1cnccc1 (Smarts matching/fingerprint pre-search)	0:04	76

[1]	SSS with pyrimidine (sketcher/fingerprint pre-search)	0:03	76

[1]	Fingerprint generation	0:13	

[1]	RGroupTable generation with Pyrimidine as core (sketcher)	0:06 (batch) 0:05 (formula)	

[1]	Jarvis Patrick clustering (generating 737 clusters)	0:19	

[1]	Sammon Map coordinate calculation	0:28	

[1]	Descriptor calculation (XLogP)	0:08	

[2]	SSS with Cc1cncnc1 (Smarts matching)	5:32	349

[2]	SSS with 5-MePyrimidine (sketcher)	1:55	486 (includes cc1cncnc1 as well as Cc1cncnc1)

[2]	SSS with Cc1cncnc1 (Smarts matching/fingerprint pre-search)	0:21	349

[2]	SSS with 5-MePyrimidine (sketcher/fingerprint pre-search)	0:13	486

[2]	Fingerprint generation	5:01	

[2]	RGroupTable generation (on Pyrimidine subset with Pyrimidine as core; sketcher)	0:28	

[2]	Descriptor calculation (XLogP)	4:37 (batch) 4:22 (formula)	

Times refer to a 2.13 GHz laptop with 4 GB of memory running Vista/Microsoft Excel 2007.

Two datasets were used: [1]: a set containing ~1.6 k pesticidal compounds, [2]: a set containing ~27 k anti-malarial compounds.

### Technical Implementation

The main enabling program is contained in an Excel for Windows workbook (Excel 97-2003 format), EnableChemicalSpreadsheetV2.1.xls. It is written in VBA using the VBA Extensibility library which allows the program to copy code to and create code in the workbook being enabled. Most code is simply copied from EnableChemicalspreadsheetV2.1.xls but some event handling routines are created specifically for the workbook being enabled; this makes possible features such as structure pop-up upon mouse hover over chart data points for example.

The CDK and OPSIN Java libraries are accessed in one of two ways. For batch processes (such as Substructure and Similarity searching) the relevant compounds are first written to file in Smiles (SMI) file format (after an in-sheet fingerprint search if necessary). Then an executable JAR file, CDKSSWin.jar is synchronously executed. This contains a number of routines corresponding to each of the available LICSS programs and taking appropriate input/output file and other control parameters. Each of these routines creates an output file and terminates, whereupon the calling VBA processes the output file appropriately. The synchronous Jar file execution is done without a command line window through Javaw.exe and CDKSSWin.jar starts by creating a pop-up Swing progress window. In this way, the routines appear to run as part of Excel.

CDK classes are widely used within CDKSSWin.jar to provide cheminformatics methods (fingerprint generation, substructure searching etc). Where available, existing open source code was adapted to use the CDK minimising the need to rewrite algorithms (eg for Jarvis Patrick clustering and Sammon projection; see acknowledgments). Algorithms for R-Group table generation, similarity searching and diverse compound picking were written in-house.

Calls to JChemPaint, to display structure editing or structure display windows, are handled quite differently. Originally (version 1 x), the JChemPaint applet was used inside a WebBrowser control within VBA. However, this approach was not suitable for the rapid display of several structures (eg for displaying all worksheet structures). From version 2.0 onwards, a JVM is run within the Excel process space so calls to Java can be made directly, without per action initialisation or context switching overheads. Calls to Java of this type are made possible by creating C++ proxies for each Java method (contained within a single CDecl dll file, CDKInterfaceDll.dll) using JNI *via *the open-source Jace project technology [10]. The C++ proxy functions may then be declared and called directly from VBA.

In practice, after one-off Java initialisation, this approach enables extremely rapid access to Java routines directly from VBA in Excel. Thus, for example, a user can render a screen's worth of structures from Smiles in < 1 second. The same method has been used for all the new Excel formulas - for example, on a 2.13 MHz laptop with 4 GB of memory running Vista, a formula entry such as: ' = GetCDKDescriptor(C2,"XLogP",1)' will calculate the XLogP descriptor for > 100 compounds per second when copied down for a column of Smiles strings (see also Table 1).

## Conclusions

LICSS is an open source chemical spreadsheet implemented in Microsoft Excel for Windows. It uses the CDK, JChemPaint and OPSIN open source libraries to provide cheminformatics functionality. LICSS-enabled worksheets and charts are self-installing, requiring no Add-Ins or anything that requires admin privileges. Enabled sheets contain only Smiles strings (with optional compact fingerprints) to represent chemistry and do not slow down Excel's calculation routines. Structures are visualised by clicking on cells containing Smiles strings or by hovering over enabled chart sheet data points. Structures for all currently visible compounds on a sheet may be simultaneously visualised 'on-the-fly'. These features mean that LICSS is suitable for worksheets containing very large (100s of 1000s) of compounds. In addition to basic visualisation and substructure/similarity searching functionality, routines for some more advanced analysis such as Sammon projection, R-Group table creation and Jarvis Patrick clustering are provided.

## Availability and Requirements

**Project name: **excel-cdk

**Project home page: **http://code.google.com/p/excel-cdk/

**Operating system: **Windows (XP, Vista or Windows 7); Microsoft Excel for Windows (97 - 2010)

**Programming languages: **VBA, Java, C++

**Other requirements (if compiling): **Jace tools

**License: **GNU GPL v2

**Any restrictions to use by non-academics**: none

## Competing interests

The authors declare that they have no competing interests.

## Authors' contributions

KRL is owner of the excel-cdk project and is the lead developer of LICSS. JL wrote the Java/C++/VBA interface code (CDKInterfaceDll). Both authors have read and approved the final manuscript.
